# Data Dissemination of the Role of Neoadjuvant Radiation in Retroperitoneal Sarcoma: A CTOS and CSSO Survey

**DOI:** 10.3390/curroncol30060434

**Published:** 2023-06-14

**Authors:** Sarah Corn, Carolyn Nessim, Christina L. Roland, Alessandro Gronchi, Carolyn Freeman, Sinziana Dumitra

**Affiliations:** 1 Department of Surgical Oncology, McGill University Health Centre, Montreal, QC H4A 3J1, Canada; 2 Department of Surgery, University of Kansas School of Medicine, Wichita, KS 67214, USA; 3 Department of Surgery, The Ottawa Hospital and Research Institute, University of Ottawa, Ottawa, ON K1H 8L6, Canada; 4 Department of Surgical Oncology, The University of Texas MD Anderson Cancer Center, Houston, TX 77030, USA; 5 Department of Surgery, Fondazione IRCCS Istituto Nazionale Dei Tumori, 20133 Milan, Italy; 6 Department of Radiation Oncology, McGill University Health Centre, Montreal, QC H4A 3J1, Canada

**Keywords:** retroperitoneal sarcoma, neoadjuvant radiation, STRASS trial, knowledge dissemination

## Abstract

Consensus guidelines call for complete resection of retroperitoneal sarcoma with consideration of neoadjuvant radiation for curative-intent treatment. The 15-month delay from the initial presentation of an abstract to the final publication of the STRASS trial results assessing the impact of neoadjuvant radiation led to a dilemma of how patients should be managed in the interim. This study aims to (1) understand perspectives regarding neoadjuvant radiation for RPS during this period; and (2) assess the process of integrating data into practice. A survey was distributed to international organizations including all specialties treating RPS. Eighty clinicians responded, including surgical (60.5%), radiation (21.0%) and medical oncologists (18.5%). Low kappa correlation coefficients on a series of clinical scenarios querying individual recommendations before and after initial presentation as an abstract indicate considerable change. Over 62% of respondents identified a practice change; however, most also noted discomfort in adopting changes without a manuscript available. Of the 45 respondents indicating discomfort with practice changes without a full manuscript, 28 (62%) indicated that their practice changed in response to the abstract. There was substantial variability in recommendations for neoadjuvant radiation between the presentation of the abstract and the publication of trial results. The difference in the proportion of clinicians describing comfort with changing practice based on the presentation of the abstract versus those that had done so shows that indications for proper integration of data into practice are not clear. Endeavors to resolve this ambiguity and expedite availability of practice-changing data are warranted.

## 1. Introduction

The rarity and diversity of sarcoma makes it a complex topic to research. Retroperitoneal sarcomas (RPSs) encompass 15% of all soft tissue sarcomas and 0.07% of all cancers. Management recommendations are primarily based on consensus guidelines put forth by organizations such as the Transatlantic Australasian Retroperitoneal Sarcoma Working Group (TARPSWG, [1]) and the National Comprehensive Cancer Network (NCCN, [2]). Multi-institutional collaborations and concerted international collaborations in the field over the last decade have led to relevant, high-quality research specifically addressing the management of RPS.

Surgery is the cornerstone of curative-intent treatment of RPS. Baseline tumor characteristics including histopathologic subtype affect the probability of local recurrence. Several reviews on neoadjuvant radiation have shown some benefit in the management of RPS, but these reviews are all based on retrospective data [2,3,4,5]. Neoadjuvant radiation therapy (RT) has been proposed to sterilize microscopically positive margins and decrease the probability of local recurrence [6]. A large review of the 1998–2011 National Cancer Database identified a higher rate of R0 resections when neoadjuvant RT was utilized, although there was no improvement in 5-year overall survival. An exploratory analysis found a small but significant improvement in 5-year survival with neoadjuvant RT in patients with high-grade tumors [7]. A propensity-score-matched study also using the NCDB compared preoperative RT to surgery alone and postoperative RT to surgery alone and found a survival benefit with radiation in this analysis [8]. In contrast, a retrospective review from eight centers in the US showed no benefit in overall survival or local recurrence in retroperitoneal sarcomas treated with either neoadjuvant or adjuvant radiation therapy [9]. Another retrospective study of well-differentiated and dedifferentiated primary retroperitoneal liposarcomas put forth by the TARPSWG found better local control with perioperative radiation therapy; however, this benefit was not identified after propensity score matching was completed [10].

The STRASS trial (EORTC 62092) has been eagerly awaited as the first successful international, RPS-specific, randomized controlled trial assessing the impact of preoperative radiotherapy on abdominal-recurrence-free survival. The initial data presented at the American Society of Clinical Oncology Annual Meeting in 2019 demonstrated no significant advantage of preoperative radiotherapy followed by surgery when compared to surgery alone in 3-year abdominal-recurrence-free survival (HR 1.01, *p* = 0.954). The STRASS trial was published 15 months later in September 2020 [11]. Given the practice-changing implications of the STRASS trial, this interval left the quandary as to how patients should be managed in the interim.

When practice-changing data are presented at ASCO, often the uptake of those changes happens rapidly even prior to full publication. As mentioned above, many studies prior to STRASS suggested a benefit of neoadjuvant radiation in RPS, and thus the negative results of this first-ever RCT were bound to create some uncertainties as to how to interpret the results prior to seeing the full publication. The delay between ASCO presentation and publication at times can be long with a risk of final results being different than the abstract presentation, thus posing a potential risk to patient safety and oncologic outcomes. This survey was important to see how other sarcoma experts were interpreting the results of the STRASS abstract presentation to clarify some of these uncertainties as well as to evaluate how knowledge and data are disseminated when they are significantly practice-changing, and whether or not this should be done with some caution. 

The purpose of the current study is twofold. First, we aim to address perspectives regarding neoadjuvant RT in the treatment of RPS during this interim period prior to publication of the data. Second, we discuss the appropriate pathway for knowledge dissemination and integration of data into clinical oncology practice.

## 2. Materials and Methods

A survey of 12 questions was developed and distributed electronically to organizations with membership of all subspecialties involved in the treatment of RPS (Connective Tissue Oncology Society, Canadian Society of Surgical Oncology). Any member from these societies could respond. There were no specific exclusion criteria. The survey was distributed via the online platform SurveyMonkey © in August 2020 and closed in September 2020 prior to publication of the STRASS trial. To increase the reach of this survey, leaders in the field also shared the survey link and reminders on social media. Reminders were sent twice at 3-week intervals to invite participants to take part in the survey. Surveys are a valid form of qualitative research frequently used in the social and psychological sciences [12].

The initial questions addressed demographics as well as the personal and institutional experience of the respondent. Clinical scenarios were presented to establish the likelihood of recommending neoadjuvant radiotherapy, each with a 5-point Likert-scaled response ranging from very unlikely to very likely. These clinical scenarios described real-life cases selected from multidisciplinary tumor board presentations. Participants were asked to indicate specific histologies that would warrant neoadjuvant radiotherapy, and a free-text response was allowed. A series of questions sought to establish levels of comfort with adopting practice changes based on abstracts or conference proceedings alone, and if practices had changed since STRASS was first presented. 

The results are presented using descriptive statistics. The association between personal or institutional factors and radiotherapy recommendations or attitudes toward change was sought. Fisher’s exact test was used to test association with categorical variables. A kappa correlation coefficient was used to calculate the correlation between pre- and post-abstract neoadjuvant radiation recommendations based on histology. The lower the correlation coefficient, the more the recommendation changed after the publication. This is a nondirectional statistic indicating change only. The data were analyzed using STATA 12 (Statacorp, College Station, TX, USA). 

## 3. Results

### 3.1. Demographics

Eighty respondents completed the survey worldwide. The demographic and institutional experience data of respondents are listed in Table 1. The survey was completed by all clinical specialties, with the largest portion consisting of surgical oncologists. Institutional and personal experience varied greatly, but almost all respondents (93%) practiced in a tertiary or quaternary referral center. Nearly 90% of respondents had access to and routinely presented cases at a multidisciplinary tumor board.

### 3.2. RT Recommendations

Responses to clinical scenarios querying the likelihood of recommending neoadjuvant radiation therapy in the interim period are listed in Table 2. Comparisons of the first two cases of well-differentiated liposarcoma with and without involvement of the femoral nerve indicate that involvement of a critical structure was related to a higher likelihood of recommending neoadjuvant radiotherapy (likely or very likely, 70% with femoral nerve involvement vs. 30% without, *p* < 0.001). Although no direct comparisons were presented for other histologies, tumors with threatened margins (scenario 3 and scenario 5) also exhibited higher frequencies of recommendations for neoadjuvant radiation.

Respondents were queried about which histologies would warrant recommendation for neoadjuvant radiotherapy both before and after the STRASS abstract was made available (Table 3). Following the ASCO presentation, only 38.8% of respondents indicated that they would recommend neoadjuvant radiotherapy for leiomyosarcoma compared to 68.8% prior to presentation, resulting in a minimal level of agreement and indicating that changes in recommendation were common. Responses to liposarcoma and solitary fibrous tumor histologies resulted in a weak level of agreement. Recommendations in angiosarcoma changed the least and retained a moderate level of agreement. Of note, the angiosarcoma subtype was not included in the STRASS trial.

In a free-text question asking for specific indications for neoadjuvant radiation, high-risk margins (3 of 11 responses) and high-grade tumors (2 of 11 responses) were mentioned in addition to undifferentiated pleomorphic sarcoma (3/11), myxoid liposarcoma (2/11) and malignant peripheral nerve sheath tumor (1/11).

### 3.3. Data Dissemination and Practice Change

Several questions were directed at the number and quality of data necessary to stimulate practice change. Only 20% of respondents were comfortable with adopting practice changes without a full manuscript available for review. Most respondents, regardless of level of experience, were uncomfortable with adopting changes based on an abstract alone, except for those who had been practicing medicine more than 20 years, who were equally comfortable and uncomfortable (Table 4). No respondents indicated that they were very comfortable with making practice changes in this situation.

Despite this, 62.5% of respondents indicated that publication of the STRASS abstract had changed practice, but most respondents also indicated discomfort in adopting changes without a full manuscript available. Of the 45 respondents who stated they were uncomfortable or very uncomfortable with making a practice change without a full manuscript, 28 (62%) indicated that practice had changed in response to the abstract. Conversely, 7 of 16 (43.8%) who responded they felt comfortable with a practice change had not yet done so. No respondent was very comfortable with a practice change based on the abstract alone.

The number of RPS cases per institution did not have an impact on the likelihood of recommending neoadjuvant radiation therapy following the STRASS presentation (*p* = 0.745) or on attitudes towards practice change without a manuscript (*p* = 0.7). All clinical specialties indicated that they had made a change in recommendations since the abstract was made available. There was no association of clinical specialty when compared to practice changes made since the STRASS presentation (*p* = 0.126) or willingness to make practice changes without a full manuscript (*p* = 0.251; Table 5).

## 4. Discussion

The rarity of retroperitoneal sarcoma combined with the diversity of histologic subtypes and corresponding clinical behaviors makes the study of this topic complex. This survey aims to highlight the urgency and necessity of not only pursuing new avenues of research but also a systematic approach at disseminating the knowledge promptly so that it can be appropriately integrated into clinical practice.

Quality publication is time-consuming. Previous studies report that the median interval from the presentation of original research at the American Society of Clinical Oncology annual meeting to manuscript publication was 2.6 years, which is substantially higher than the time needed for the STRASS manuscript (1.2 years). Importantly, however, only 77% of these presentations had been published at all within 5 years [13], which is consistent with other measures of publication delay [14,15]. This lag is partly due to the pace of the peer-review process. The medical community relies on the peer-review system to judge scientific quality and to protect us and our patients from falling victim to fraudulent or poor-quality science and inaccuracies [16]. It takes more than 100 days to publish the final result after completing peer review after the manuscript has been accepted [17]. 

These issues are compounded with nonpublication. Without publication, data are not included in reviews and meta-analyses, which can lead to an over- or underestimation of treatment effects and skew clinical recommendations [18]. Publication bias with selective publication of studies with positive results has been clearly identified in many medical fields, including oncology [19,20]. Negative trials compose 71% of the unpublished presentations in the study of ASCO presentations by Tam et al. [14]. Those that were selected as plenary or oral presentations were significantly more likely to be published compared to those listed in the conference proceedings only (*p* = 0.03) [21]. Negative results face more of a delay when published (407 days, 95% CI 298–705 days vs. 272 days, 95% CI 211–318 days; *p* < 0.001) [22]. A review of unpublished trials evaluated by expert oncologists and hematologists estimated that none of them were expected to have a critical impact on clinical decisions; however, 32 (59%) were estimated to have had some impact on clinical decision making if results were made available at the time [14]. 

Critical evaluation of data prior to adopting practice-changing concepts is key. Oncology practice guidelines have utilized conference abstracts [23,24]; however, discrepancies in results from conference presentation to publication should be considered. Reported results of primary end points are consistent in 59–78% of publications, with final conclusions remaining consistent in 70–93% [25,26,27,28]. Another evaluation showed substantial variability in data with only 58% of published trials documenting primary end points within 5% of that included in the published abstract, with this number decreasing to 11% in the abstracts indicating “interim results” [22]. Fortunately, the statistical significance and conclusions remained unchanged in 89% and 91% of comparisons, respectively [22]. However, in those cases where conclusions are different, adopting abstract results can cause risk to patient safety and oncologic outcomes. These discrepancies clearly call for caution in the early adoption of practice-changing information. 

Our survey aimed to assess attitudes toward recommending neoadjuvant radiotherapy in the interim period prior to full publication of the STRASS trial results. Responses to the clinical scenarios were diverse, with evidence that recommendations did change in response to the STRASS presentation for some histologies, most notably in leiomyosarcoma. It is plausible that the ASCO presentation introduced variability, with some clinicians adhering to the current guidelines and others adopting changes based on the presentation. Only 20% of survey respondents indicated that they were comfortable adopting practice changes without a full manuscript available for review; however, a substantially higher proportion (62.5%) indicated that their practice had changed in some way in response to the conference presentation. Clinicians may have been hesitant to make any practice changes given that a trend towards benefit with the addition of preoperative radiotherapy was suggested at the ASCO presentation for liposarcoma subgroups, which comprised a large proportion of the STRASS study population. Furthermore, some respondents indicated that high-grade histology would be a prompt to recommend neoadjuvant RT. However, the results of the STRASS trial indicate that benefit may be derived from RT in low-grade but not high-grade tumors. These data imply that we, as physicians, may not have a firm concept of how and when to apply practice-changing data prior to manuscript publication. This study identified no significant relationships when comparing individual clinical (e.g., years of experience, clinical specialty) or institutional (e.g., RPS case volume, location, type of practice) experience that would predict those more likely to change practice recommendations without a full manuscript. In situations such as this, where impactful practice-changing data should produce action, the current system may be limiting. Several strategies have been proposed to maximize efficiency in these critical situations, such as preprinting, posting full conference presentations and making funding contingent on publication. While not perfect, this would provide more information and allow for more critical evaluation of actionable data while awaiting publication. Notably, some practitioners might feel the need to wait for the long-term results prior to making changes in their practice. This study had several strengths. To our knowledge, this is the first study to evaluate the integration of data into clinical practice in surgical oncology and specifically for RPS. This is novel as it is not often evaluated, as the “real world” actual uptake of practice change occurs after a presentation at ASCO. This survey benefitted from international participation from all clinical specialties. The clinical scenarios were based on real-life cases and are reflective of practice, increasing the applicability of the results. 

This study is subject to the limitations inherent to any survey, particularly in that respondents may have also participated in the STRASS trial and co-written the manuscript and thus could have been biased by prior knowledge of the results. Moreover, the survey did not evaluate the respondents’ level of exposure to the abstract results of STRASS at ASCO; those who were present at ASCO and part of the question-and-answer period and those reading the abstract later on in isolation may have had a very different interpretation of the results, which would have had a significant impact on their comfort level of adopting changes in their practice. Surgeons were overrepresented in this population. Moreover, a pilot questionnaire was not assessed for clarity, and biases of the authors could have been introduced in the formulation of questions. This non-validated survey was distributed electronically via email and social media advertisements. While this method potentially reaches a larger audience, it is impossible to know the true response rate, although it is reported to be lower with this form of distribution [29], and the absolute number of respondents is limited (n = 80). Bias may be introduced if differences exist in those likely to respond to a social media post or not. Furthermore, there are many other clinical scenarios that could have been asked in the survey, including more histological subtypes or the impact of neoadjuvant chemotherapy on the selection of radiation. There is some evidence, however, that if a survey is too long, there are fewer respondents and the quality of responses decreases [30,31]. Furthermore, the study team felt the survey questions should reflect the same patient population as that included in the STRASS trial. The trial included resectable patients that had mainly liposarcoma and leiomyosarcoma (only 11 patients in the “other” category) and was focused on neoadjuvant radiation regardless of chemotherapy. In fact, patients were excluded from the trial if they had chemotherapy, and thus the survey questions reflected this. The survey included the most common clinical scenarios frequently seen in the treatment of RPS, of the five most common histologies found in the retroperitoneum. 

Further studies could provide surveys on a critique of the STRASS trial results and more qualitative interviews on how clinicians have interpreted this trial and how/if it has affected their practice, especially with the release of the STREXIT trial [32] (patients that were not randomized to STRASS), showing the potential benefit of neoadjuvant radiation in WDLPS and G1-2 DDLPS and continuing to show no benefit in G3 DDLPS and LMS. This study team, as a future prospective, has planned a follow-up survey now that STRASS has been formally published, not only as an abstract form, to see if the responses change once again post-publication. 

In conclusion, this survey study underscores the need for prompt reporting of actionable, practice-changing data. Further data are needed, and it will be insightful to compare the results of this survey to one completed after STRASS publication to assess the practice changes it has brought about.

## Figures and Tables

**Table 1:** Demographics and institutional experience.

Respondent Data	% of Respondents
**Specialty**	
Surgical oncology	60.5%
Radiation oncology	21.0%
Medical oncology	18.5%
**Years of practice**	
<5 years	23.5%
5 to 10 years	29.6%
10 to 20 years	29.6%
>20 years	17.3%
**Routine presentation at multidisciplinary tumor board**	
Yes	88.9%
No	11.1%
**Annual volume of RPS cases**	
None	2.5%
1 to 10	17.3%
11 to 25	35.8%
26 to 50	22.2%
**Practice type**	
Community hospital/system	7.4%
Tertiary or quaternary academic referral center	92.6%
**Practice location**	
North America	62.9%
South America	1.4%
Europe	22.9%
Australasia	12.9%

**Table 2:** Likelihood of recommending neoadjuvant radiotherapy for certain clinical scenarios following presentation of the STRASS abstract.

Scenario	Very Unlikely	Unlikely	Neutral	Likely	Very Likely
A healthy 50-year-old female presents with a biopsy-proven 16 cm well-differentiated liposarcoma.	25%	26.3%	18.8%	18.8%	11.3%
A healthy 50-year-old female presents with a biopsy-proven 16 cm well-differentiated liposarcoma encasing the femoral nerve.	15.0%	6.3%	8.8%	35.0%	35.0%
A healthy 60-year-old female presents with a heterogenous 25 cm mass in the left retroperitoneum abutting the kidney and tail of pancreas. A core needle biopsy of the solid component confirms dedifferentiated liposarcoma.	7.5%	21.3%	12.5%	35.0%	23.8%
A healthy 60-year-old male presents with a biopsy-proven 7 cm leiomyosarcoma arising from the left external iliac vein with extensive collateralization.	18.8%	28.8%	15.0%	25.0%	12.5%
A healthy 68-year-old male presents with a 12 cm pelvic malignant solitary fibrous tumor sarcoma abutting the sacrum.	15.5%	18.8%	15.0%	31.3%	20.0%

**Table 3:** Likelihood of recommending neoadjuvant radiotherapy for histologic subtypes prior to and after the presentation at ASCO.

Histology	Recommend Neoadjuvant Radiation		Kappa Correlation Coefficient
	Prior to Presentation (%)	After Presentation (%)	
Leiomyosarcoma	55 (68.8)	31 (38.8)	0.35
Dedifferentiated liposarcoma	62 (77.5)	56 (70.0)	0.42
Well-differentiated liposarcoma	23 (28.8)	35 (43.8)	0.52
Solitary fibrous tumor	39 (48.8)	28 (35.0)	0.57
Angiosarcoma	41 (51.3)	26 (32.5)	0.63

**Table 4:** Adoption of practice changes based on years of experience.

Years in Practice	Uncomfortable	Neutral	Comfortable
<5 years	55.5%	33.3%	11.1%
5 to 10 years	62.5%	20.8%	16.7%
10 to 20 years	58.3%	25.0%	16.7%
>20 years	42.9%	14.3%	42.9%

**Table 5:** Attitudes toward adopting practice changes based on abstract/oral presentation alone.

Clinical Specialty	Uncomfortable	Neutral	Comfortable
Medical oncology	40.0%	20.0%	40.0%
Radiation oncology	65.0%	17.7%	17.7%
Surgical oncology	58.3%	23.8%	14.6%

## Data Availability

Not applicable.
